# Severe hypertrophic cardiomyopathy in a patient with a homozygous MYH7 gene variant

**DOI:** 10.1016/j.heliyon.2022.e12373

**Published:** 2022-12-16

**Authors:** Walter Serra, Giulia Vitetta, Vera Uliana, Federico Barocelli, Valeria Barili, Isabella Allegri, Diego Ardissino, Francesca Gualandi, Antonio Percesepe

**Affiliations:** a Cardiology Division, University Hospital of Parma, Italy; b Medical Genetics Unit, University Hospital of Parma, Italy; c Medical Genetics, Department of Medicine and Surgery, University of Parma, Italy; d Neurology Unit, University Hospital of Parma, Italy; e Medical Genetics Service, Department of Mother and Child, University Hospital S. Anna, Ferrara, Italy

**Keywords:** Hypertrophic cardiomyopathy, Imaging, Genetic analysis

## Abstract

**Background:**

Hypertrophic cardiomyopathy is an autosomal dominant disease. The main feature of this disorder is its occurrence in patients who present a left ventricular hypertrophy, unexplained by the loading conditions, usually asymmetric with greatest involvement most commonly of the interventricular septum.

Case presentation During a sports medicine control, a ultrasound scan in a 17 years old patient has shown a concentric left ventricular parietal hypertrophy associated with a 23 mm mid- basal interventricular septum thickness. After genetic counselling, a positive family history for hypertrophic cardiac disease and parents’ consanguineity was found. The genetic basis of the hypertrophic cardiomyopathy was investigated through a dedicated gene panel. The genetic test has revealed the presence of the variant c.3424G>A (p.Glu1142Lys) in the MYH7 gene in a homozygous state. Genotyping of the parents and of the two brothers revealed the presence of the MYH7 variant in heterozygosity in both parents and in the younger brother. In all of them, variable signs of hypertrophic cardiomyopathy were found.

**Conclusions:**

Our findings report the presence of a homozygous variant in a sarcomeric gene (MYH7) which gave rise to early HCM, whereas the variant in a heterozygous state was associated to much milder cardiac phenotypes in the affected relatives. The onset and the progression of the hypertrophic cardiomyopathy in the reported family is to be referred to the presence of the variant in hetero- or homo-zygosity in a gene dosage manner.

## 1 Introduction

Defects in sarcomeric proteins are responsible of a wide phenotypic spectrum spanning from mild hypertrophic cardiomyopathy (HCM) [1], to generalized muscular disorders with variable severity and age of onset. Thousands of variants, mostly private, have been identified in sarcomeric genes, most commonly in *MYH7* (β-Myosin heavy chain) and *MYBPC3* (myosin binding protein C) both encoding for heavy chain proteins. Altogether, *MYH7* and *MYBPC3* account for approximately half of the patients with familial HCM [2, 3]. Mutations in *TNNT2*, *TNNI3* (encoding for Cardiac troponin T and I), *TPM1 (*α-Tropomyosin*), ACTC1* (cardiac α-actin), *MYL2* (myosin light chain 2), *MYL3* (myosin light chain 3) and *CSRP3* (Cysteine and Glycine Rich Protein 3) are less common causes of HCM.

HCM is highly variable in its clinical expression and outcome, even for individuals belonging to the same family: clinical manifestations can vary greatly, from asymptomatic to severe forms, or even to sudden cardiac death (SCD), which may be the first sign of the disease. The main clinical feature of the disease is a cardiac hypertrophy, unexplained by the loading conditions, a non-dilated left ventricle and a normal or increased ejection fraction. It commonly involves the basal interventricular septum subjacent to the aortic valve, whereas other myocardial regions, such as the apex, the mid-portion as well as the posterior wall of the left ventricle are occasionally reported. After the clinical diagnosis, genetic testing is meant to establish a genetic etiology of the disease in the proband and to identify the relatives at risk of developing the disease [4, 5, 6].

About 5% of patients affected by HCM carry biallelic gene variants and show more severe clinical features [7, 8]. For example, homozygous variants in MYH7 cause a myosin storage myopathy (MIM 255160), featured by a hyaline-like accumulation of myosin in muscle fibres [9], whose clinical features include hypotonia, scapuloperoneal or generalized muscle weakness with a disease onset ranging from the neonatal period to adulthood and a slow progression. In the reported cases also early-onset spinal rigidity with scoliosis requiring spinal fusion and progressive respiratory impairment with a non-invasive ventilation with preserved ambulation have been described [10, 11].

## 2 Clinical presentation

**Figure 1:**
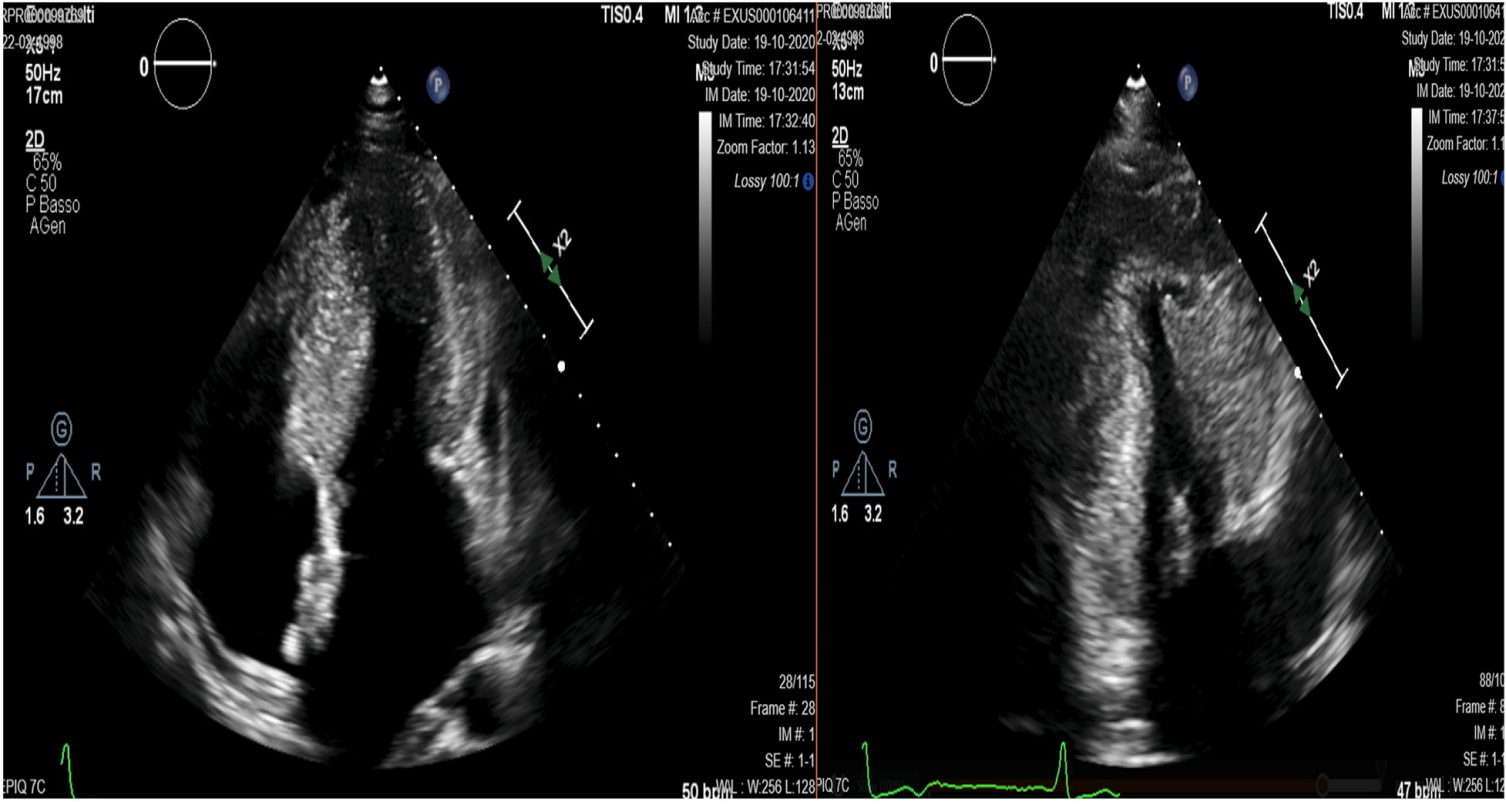
Ultrasound scan shows a concentric left ventricular parietal hypertrophy associated with a 23 mm mid- basal interventricular septum thickness.

**Figure 2:**
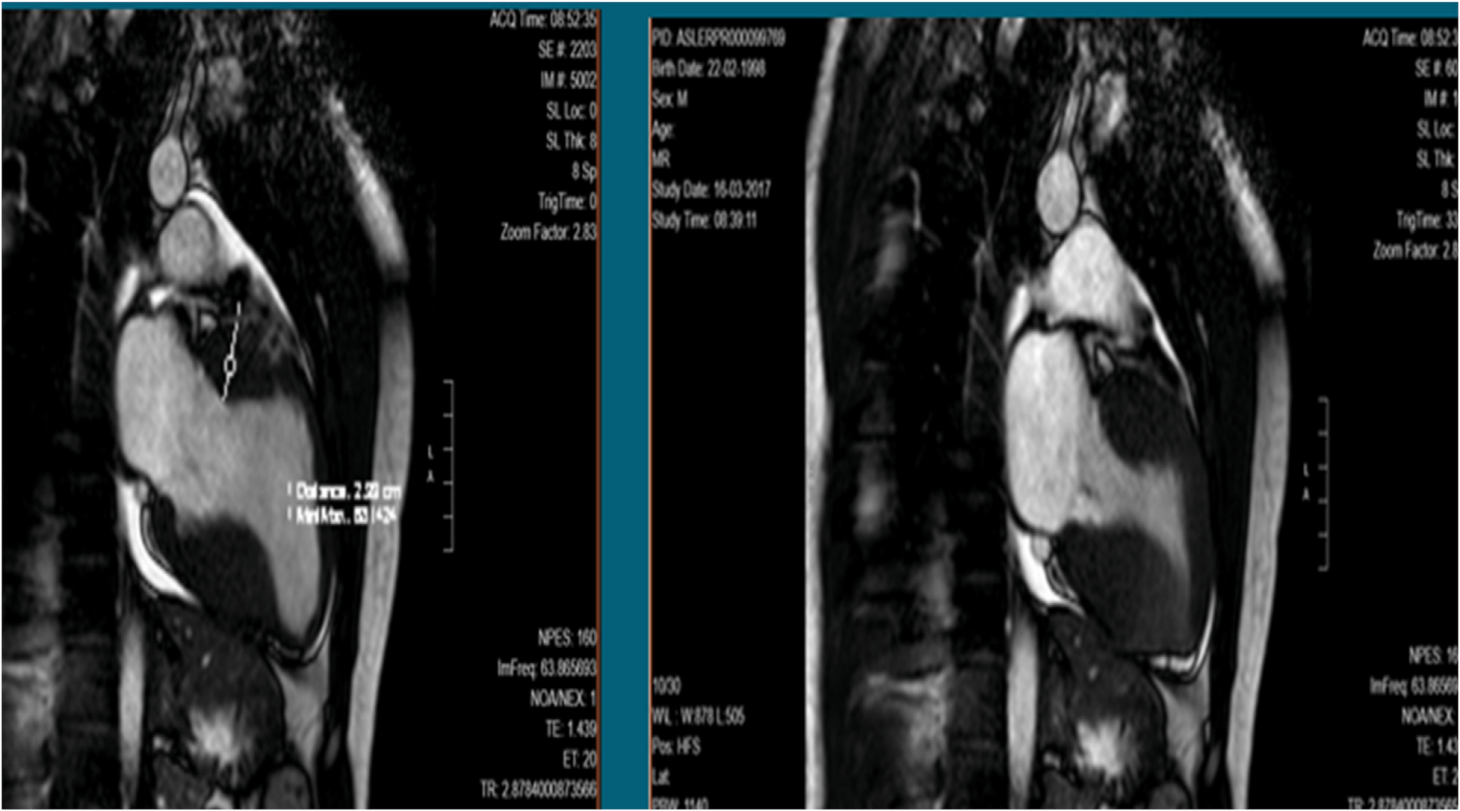
MRI reveals an evolving condition with an increased mid-basal parietal thickness up to 23–25 mm compared to the previous exam, with fibrosis in the inferior septal wall.

**Figure 3:**
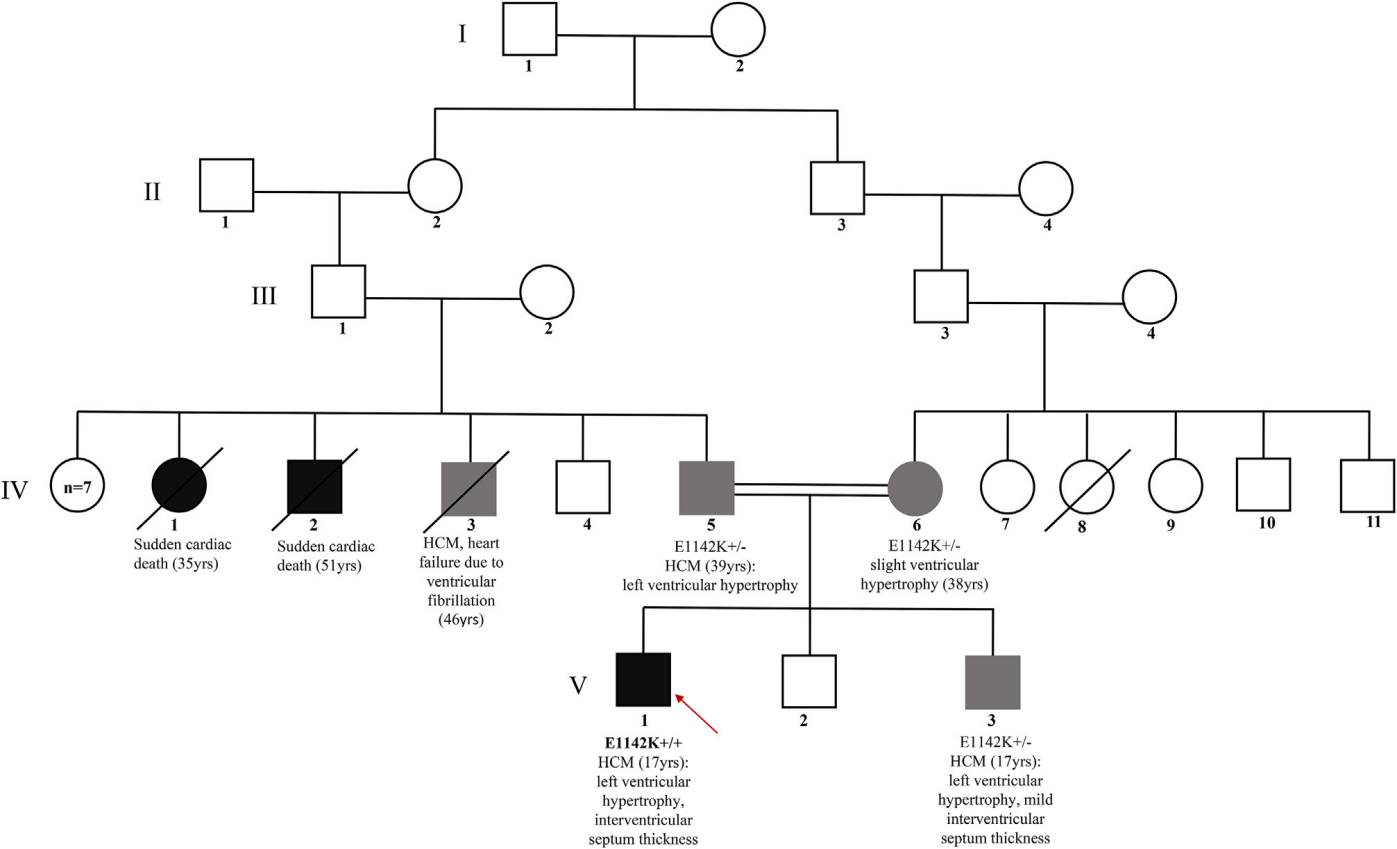
Family pedigree with clinical and genetic features if available.

During a sports medicine echocardiographic assessment at 17 years of age, a cardiac hypertrophy, a right ventricular dilatation and an atrial septal defect (ostium secundum) were diagnosed in the proband, who after underwent a percutaneous transcatheter closure of the interatrial defect by Amplatzer Device. During the follow up he showed severe septal hypertrophy with outflow obstruction in basal conditions of 50 mm/Hg and the presence of anterior systolic movement (SAM); the global longitudinal strain was preserved. Mitral insufficiency was moderate by Venturi effect (Figure 1). After one year, a heart MRI exhibited a progression in the hypertrophy with thickening of the basal septum (23mm), infero-lateral wall (30mm) and middle septum (25mm). The maximum wall thickness (MWT) was 34 mm in the infero-septal segment, with Late Gadolinium Enhancement areas due to focal regions of fibrosis. The outcome of the closure of the interatrial defect is shown in Figure 2. Moreover, electromyography exhibited a mild muscle involvement (i.e. in gastrocnemius muscle), thus confirming the muscle involvement shown by the mild increase in serum level of creatine kinase enzymes (between 300 and 400 U/L in several measurements, normal range 60–190 U/L). The patient was then referred to the Cardiogenetic outpatient clinic where the presence of relatives with cardiac disease or sudden cardiac death have been searched, reconstructing the proband’s family tree in detail (Figure 3). Family history brought to light that one paternal uncle had heart failure due to ventricular fibrillation at 46, another had a sudden death at 51 and finally a paternal aunt deceased at 35 years of age also for a sudden death. Finally a consanguineity in the proband’s parents was revealed (second-degree cousins). The next generation sequencing (NGS) panel for hypertrophic cardiomyopathies was indicated, after informed consent.

## 3 Investigations

**Figure 4:**
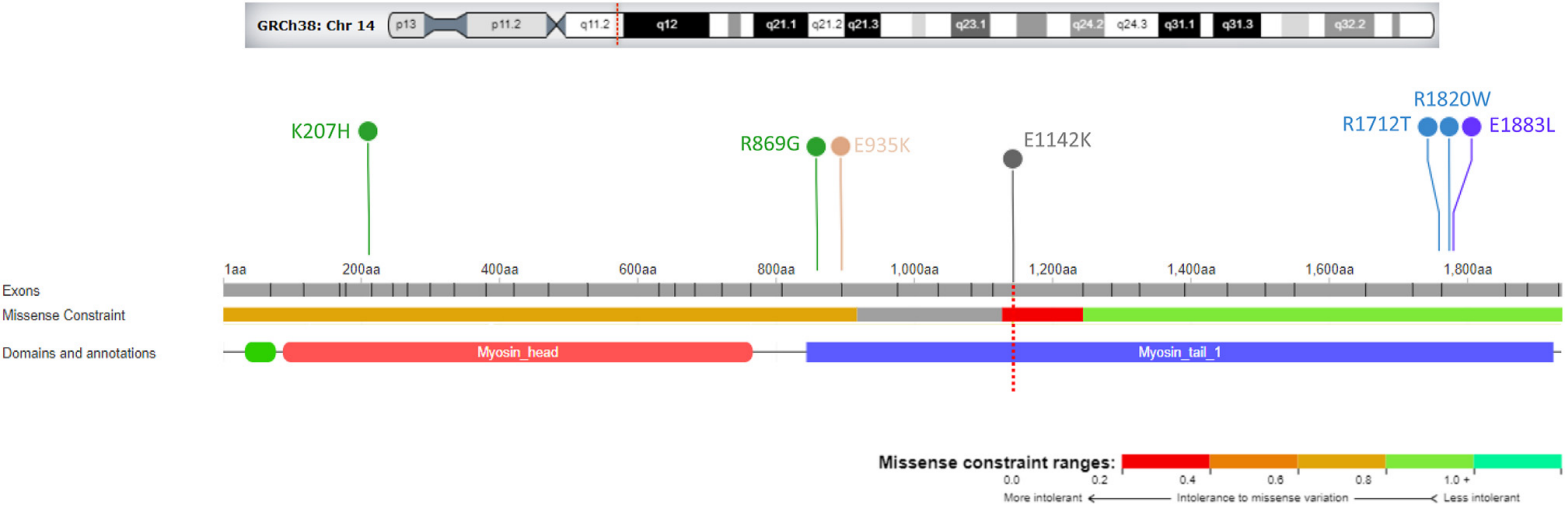
Homozygous *MYH7* genomic variants analysis.

The NGS gene panel revealed the presence of the variant NM_000257.4:c.3424G>A (p.Glu1142Lys, rs730880774) in *MYH7* in 100% of the reads, compatible with homozygosity for the variant allele. The variant, which lies in the exon 27 of MYH7, was rated as likely pathogenic according to the American College of Medical Genetics and Genomics (ACMG) criteria [12], by attributing PM2 (absent from control), PP3 (several computational analysis), PP1 (the segregation criteria) and PP4 (the phenotype match). Moreover, the predictive algorithms scored the variant as extremely deleterious due to its location in a highly conserved myosin tail domain (Figure 4). The familial genotyping for the variant (parents and two brothers) revealed the presence of the Glu1142Lys of *MYH7* in both father and mother and in the youngest brother, whereas the other brother did not inherit the variant. A further clinical evaluation showed that the father and the youngest child manifested a typical HCM presentation (the latter had also initial regions of LGE in the interventricular septum). A mild ventricular hypertrophy was identified in the mother. The genotype-negative brother was clinically unaffected.

## 4 Discussion

The severe hypertrophic cardiomyopathy of the reported patient has been demonstrated as associated to the presence of the homozygous variant of the sarcomeric *MYH7* gene, which, together with *MYBPC3*, is the most commonly involved gene in hyperthrophyc cardiomyopathy [5]. International databases have already reported the p. Glu1142Lys in MYH7, but in our case the study of the family history of the proband and of the segregation of the variant have allowed a more convincing attribution of the pathogenic significance of the mutation.

The pGlu1142Lys variant has been already described in a large cohort study of patients with hypertrophic cardiomyopathy [8],in which the authors displayed also homozygous patients in MYH7, suggesting that patients with multiple variants had higher risk for cardiovascular death, as well as all-cause death, SCD, and heart failure-related death, thus prospectively demonstrating that the dosage of rare variants may predict clinical outcomes.

In our proband it has been noted how the homozygous mutation has brought to a severer phenotypic and a faster evolution of the disease. HCM was diagnosed during the patient’s adolescence and the RMN results, performed a year after the previous scan, have shown a sudden deterioration of his conditions with an increase in the wall thickness (from 16mm to 23–25mm) associated to interstitial fibrosis, a clear symptom of a worsening in his cardiomyopathy. When the proband was 20 years old, the progression of the hypertrophy and the high risk for sudden cardiac death recommended an ICD placement. His parents, both carrying the variant in a heterozygous state, did not present the same severe clinical manifestations except for a mild ventricular hypertrophy in the mother and a more pronounced one in his father; the proband’s youngest brother, also a heterozygous carrier (seventeen years old when diagnosed) showed the same minor hypertrophy associated with a negligible septum thickness. The case is intriguing because HCM is an autosomal dominant disease, whereas our proband is homozygous for the *MYH7* variant. Homozygosis then results in a more deleterious phenotype compared to the heterozygous family members, as reported also by others [8]. Multiple rare variants in sarcomere genes thus represents a risk factor for adverse clinical outcome, and could be added to the risk stratification algorithm in HCM.

The variant, which lies in the exon 27 of MYH7, was rated as likely pathogenic according to the American College of Medical Genetics and Genomics (ACMG) criteria [12], by attributing PP1 (the segregation criteria), PP3 (several computational analysis), PP4 (the phenotype match) and PS4 (the prevalence of the variant in affected individuals is significantly higher than in controls).

MSMB clinical manifestations involve muscular and scapuloperoneal weakness and atrophy, scoliosis, presence of hyalin bodies in cardiac and skeletal type 1 muscle fibres. Affected patients may present cardiac symptoms, which can be variable and not always present: out of the 6 homozygous patients shown in Table 1, two of them had cardiomyopathy. The other three variants (Table 1, variant 5, 6, 7) were reported in a retrospective study [13] dealing with various double mutations affecting 8 core genes (MYBPC3, MYH7, TNNI3, TNNT2, MYL2, TPM1, ACTC1, MYL3) in an homozygous and compound heterozygous state. In these three families, as well as in our case, the heterozygous family members disclosed a milder form of cardiomyopathy, while the carriers of double mutation had a more severe clinical condition with an early-onset and a progressive cardiomyopathy towards an end stage with high arrhythmic risk. Consistently, Christian and colleagues [14] have pointed out that the mean age-of-onset in adults with multiple variants is 7.0 years earlier than those with a single variant, consequently leading to a more likely requirement of cardiac transplant.

**Table 1:** Homozygous MYH7 variants.

#	genotype	exon	ACMG	MLVWT (mm)	Phenotype of the Homozygous carrier
1	c.3424G>A p.Glu1142Lys	27	LP	34 mm	Atrial septal defect at 17 y, LV dilatation and ejection fraction at 68%. Fibrosis, severe Mitral Insufficiency. ICD implant at 19 y.
2	c.5134C>T p.Arg1712Trp	35	P	1.NA 2.NA	1. muscle weakness at age 12, severe restrictive lung disease, severe scoliosis with spinal rigidity. Normal cardiac function. 2. Intramuscular fatty replacement in the gluteal muscles. Spinal rigidity, mild restrictive lung disease at age 12, NIV at 13 y. Normal cardiac function.
3	c. 5458C>T p.Arg1820Trp	37	LP	1.NA 2.10 mm	1. Severe muscle weakness at 28 y, 40 y moderate weakness and atrophy in the shoulder and peroneal muscles, mitral valve prolapse without any cardiac symptoms 2. Severe muscle weakness at 33y, LV systolic and diastolic dysfunction. DCM. Respiratory Insufficiency, NIV
4	c.5647G>A p.Glu1883Lys	38	LP	NA	44y daytime sonnolence, leg weakness, RF,short stature, thoracic scoliosis, proximal muscle weakness, HF and biventricular HCM. NIV.
5	c.2605C>G p.Arg869Gly	22	LP	1.35 mm 2.17 mm	1. End stage progression, AF at 17 y. LV ejection fraction 80%. Pacemaker implant at 27. SAM, Possible progression to end-stage HCM at 37 y, with a regression of the hypertrophy (MWT 20mm) and a LA dilatation (85mm) 2. End stage progression, AF at 17 y. Stroke and pacemaker implant at 28y. Progressed to end-stage HCM at 33 y
6	c.619A>C p.Lys207Gln	7	P	21 mm	1.End stage progression, developed HF and AF + appropriate ICD intervention
7	c.2803G>A p.Glu935Lys	23	LP	1.26 mm 2.26 mm	1.ES progressed to end-stage dilated HCM and died of HF at 31 y 2. SCD at 34 y

1) **Patient 1** of the table refers to our case, which has been added to the list.

2) Beecroft SJ, van de Locht M, de Winter JM, Ottenheijm CA, Sewry CA, Mohammed S, Ryan MM, Woodcock IR, Sanders L, Gooding R, Davis MR, Oates EC, Laing NG, Ravenscroft G, McLean CA, Jungbluth H. Recessive MYH7-related myopathy in two families. Neuromuscul Disord. 2019 Jun;29(6):456-467. doi: 10.1016/j.nmd.2019.04.002. Epub 2019 Apr 12. PMID: 31130376.

3) Yüceyar N, Ayhan Ö, Karasoy H, Tolun A. Homozygous MYH7 R1820W mutation results in recessive myosin storage myopathy: scapuloperoneal and respiratory weakness with dilated cardiomyopathy. Neuromuscul Disord. 2015 Apr;25(4):340-4. doi: 10.1016/j.nmd.2015.01.007. Epub 2015 Jan 26. PMID: 25666907.

4) Tajsharghi H, Oldfors A, Macleod DP, Swash M. Homozygous mutation in MYH7 in myosin storage myopathy and cardiomyopathy. Neurology. 2007 Mar 20;68(12):962. doi: 10.1212/01.wnl.0000257131.13438.2c. PMID: 17372140.

5)Richard P, Charron P, Leclercq C, Ledeuil C, Carrier L, Dubourg O, Desnos M, Bouhour JB, Schwartz K, Daubert JC, Komajda M, Hainque B. Homozygotes for a R869G mutation in the beta -myosin heavy chain gene have a severe form of familial hypertrophic cardiomyopathy. J Mol Cell Cardiol. 2000 Aug;32(8):1575-83. doi: 10.1006/jmcc.2000.1193. PMID: 10900182.

6)Mohiddin SA, Begley DA, McLam E, Cardoso JP, Winkler JB, Sellers JR, Fananapazir L. Utility of genetic screening in hypertrophic cardiomyopathy: prevalence and significance of novel and double (homozygous and heterozygous) beta-myosin mutations. Genet Test. 2003 Spring;7(1):21-7. doi: 10.1089/109065703321560895. PMID: 12820698.

7) Nishi H, Kimura A, Harada H, Adachi K, Koga Y, Sasazuki T, Toshima H. Possible gene dose effect of a mutant cardiac beta-myosin heavy chain gene on the clinical expression of familial hypertrophic cardiomyopathy. Biochem Biophys Res Commun. 1994 Apr 15;200(1):549-56. doi: 10.1006/bbrc.1994.1483. PMID: 7909436.

Our findings will support physicians and geneticists with diagnosis, management and counselling of patients with recessive MYH7 disease and their relatives.

## 5 Conclusion

The presented case has shown how important the genetic screening is in order to confirm a suspicious HCM detected through diagnostic imaging. In particular, a genetic test allows finding pathogenic variants very early in patients whose phenotype has not manifested yet, as it has happened for the patient’s brother. Genetic testing also consents to optimize resources by directing only genotype positive individuals to a further clinical follow up. Our findings suggest that the presence of a homozygous variant gives rise to the onset of HCM in our patient and that the severity of the onset and progression could be attributed to the presence of multiple mutations in a gene dosage manner.

## Ethical approval and consent to participate

Not applicable.

## Consent to publication

Yes.

## Declarations

### Author contribution statement

All authors listed have significantly contributed to the investigation, development and writing of this article.

### Funding statement

This research did not receive any specific grant from funding agencies in the public, commercial, or not-for-profit sectors.

### Data availability statement

Data will be made available on request.

### Declaration of interests statement

The authors declare no conflict of interest.

### Additional information

No additional information is available for this paper.
